# The Los Angeles County Community Disaster Resilience Project — A Community-Level, Public Health Initiative to Build Community Disaster Resilience

**DOI:** 10.3390/ijerph110808475

**Published:** 2014-08-19

**Authors:** David Eisenman, Anita Chandra, Stella Fogleman, Aizita Magana, Astrid Hendricks, Ken Wells, Malcolm Williams, Jennifer Tang, Alonzo Plough

**Affiliations:** 1 Los Angeles County Department of Public Health Emergency Preparedness and Response Program, 600 S. Commonwealth Avenue, Suite 700, Los Angeles, CA 90005, USA; E-Mails: sfogleman@ph.lacounty.gov (S.F.); aimagana@ph.lacounty.gov (A.M.); ahendricks@ph.lacounty.gov (A.H.); 2 Center for Public Health and Disasters, UCLA Fielding School of Public Health, P.O. Box 951772, Los Angeles, CA 90095, USA; 3 RAND Corporation, 1200 South Hayes Street, Arlington, VA 22202, USA; E-Mail: chandra@rand.org; 4 Center for Health Services and Society, David Geffen School of Medicine, 10920 Wilshire Boulevard, Suite 300, Los Angeles, CA 90024, USA; E-Mails: kwells@mednet.ucla.edu (K.W.); j1tang@ucla.edu (J.T.); 5 RAND Corporation, 1776 Main Street, Santa Monica, CA 90401, USA; E-Mail: malcolm_williams@rand.org; 6 Robert Wood Johnson Foundation, Route 1 and College Road East, P.O. Box 2316, Princeton, NJ 08543, USA; E-Mail: aplough@rwjf.org

**Keywords:** resilience, disaster, preparedness, public health, randomized trial, community engagement, organizational linkage, table-top exercise

## Abstract

Public health officials need evidence-based methods for improving community disaster resilience and strategies for measuring results. This methods paper describes how one public health department is addressing this problem. This paper provides a detailed description of the theoretical rationale, intervention design and novel evaluation of the Los Angeles County Community Disaster Resilience Project (LACCDR), a public health program for increasing community disaster resilience. The LACCDR Project utilizes a pretest–posttest method with control group design. Sixteen communities in Los Angeles County were selected and randomly assigned to the experimental community resilience group or the comparison group. Community coalitions in the experimental group receive training from a public health nurse trained in community resilience in a toolkit developed for the project. The toolkit is grounded in theory and uses multiple components to address education, community engagement, community and individual self-sufficiency, and partnerships among community organizations and governmental agencies. The comparison communities receive training in traditional disaster preparedness topics of disaster supplies and emergency communication plans. Outcome indicators include longitudinal changes in inter-organizational linkages among community organizations, community member responses in table-top exercises, and changes in household level community resilience behaviors and attitudes. The LACCDR Project is a significant opportunity and effort to operationalize and meaningfully measure factors and strategies to increase community resilience. This paper is intended to provide public health and academic researchers with new tools to conduct their community resilience programs and evaluation research. Results are not yet available and will be presented in future reports.

## 1. Introduction

As disasters increase in scale, frequency, length, and costs worldwide it is apparent that communities cannot rely on national governmental dollars and agencies to ensure effective and comprehensive disaster response and recovery. Also, disasters in urban centers with diverse communities and growing inequalities challenge governmental capabilities to handle the complex social, health, housing, and financial challenges of response and recovery without local, community involvement [1]. In preparation for the post-2015 Hyogo Framework for Action, countries across the globe emphasize that local governments and community organizations must be supported and encouraged to implement community resilience programs [2]. In the United States, local health departments and responder agencies have often turned to non-governmental agencies and local community and faith based organizations during disasters for their knowledge of needs, resources and social complexities in the neighborhoods they serve [3,4,5,6]. 

Community resilience, or the sustained ability of a community to withstand and recover from adversity, emphasizes that effective and efficient disaster risk reduction, response and recovery requires a whole of community approach, specifying that partnerships with nongovernmental partners, engagement of local communities and orientation to community self-sufficiency is the foundation of this approach [7]. The World Health Organization urges member states to use coordinated, multisectoral approaches to disaster risk reduction, response and recovery [8]. In the United States, community resilience has become integral to several national directives [9,10], including the Center for Disease Control (CDC) and Prevention’s public health emergency preparedness (PHEP) cooperative agreements [11] and the Federal Emergency Management Agency’s (FEMA) “Whole of Community Planning” imperative [12]. FEMA’s approach stipulates as policy that collaboration, local empowerment and collective community response are central to preparedness, response and recovery [13]. “The CDC PHEP agreements that give funding to state and local health departments provide a set of public health preparedness capabilities to assist health departments with their strategic planning for improving preparedness and creating more resilient communities” [11]. Capability 1 specifically addresses community resilience in the area of community preparedness. It includes determining community health risks, identifying vulnerable or at-risk populations and those with access and functional needs, building community partnerships, engaging community organizations to foster public health social networks, and coordinating training to enhance engagement of lay persons in community resilience. 

While these mandates support preparedness activities at the community level, there are few evidence based methods to building community resilience in the United States [14]. It is unclear what local public health should do or the role it should play to build community resilience [15]. Public health officials faced with operationalizing the CDC capabilities need both evidence-based strategies for implementing community disaster resilience and evaluation methods for measuring results. 

This paper describes the theoretical rationale, intervention design and evaluation of a public health program for increasing community resilience (CR) in selected neighborhoods in a major, urban center. The Los Angeles County Community Disaster Resilience (LACCDR) Project is providing the opportunity to translate a theory of community resilience building into practice, strengthen disaster resilience in Los Angeles County communities, and to evaluate its outcomes. The goal of the LACCDR Project is to increase the readiness of communities and the people who live in them to prepare for, respond to, and recover from a natural disaster, other emergency, or public health event through a community-based approach. This paper is intended to provide public health and academic researchers with new tools to conduct their community resilience programs and evaluation research.

## 2. Methods 

### 2.1. Intervention Development 

As previously reported, the LACCDR project was developed over a period of two years by the Los Angeles County Department of Public Health working closely with community, academic, government and business partners [16,17]. During 2010–2012, the project engaged a broad array of community stakeholders representing government agencies and community-based organizations to identify and develop strategies that would bolster resilience. The project engaged these stakeholders through community forums, working groups, and community surveys that led to the design of the LACCDR Project [17].

### 2.2. Collaboration with Diverse Stakeholders

The foundation of the LACCDR Project is collaborations between public health, several academic institutions (UCLA, RAND Corporation, Loma Linda University, USA), governmental and non-governmental agencies (United States Geological Survey Science Application for Risk Reduction, Emergency Network of Los Angeles, USA), businesses (private media consultants) and the communities themselves. These stakeholders comprise the LACCDR Steering Committee that guides the implementation and evaluation of the project. The Project is funded by the CDC PHEP grant with supplemental funding provided by the National Institutes of Mental Health and the Robert Wood Johnson Foundation. 

### 2.3. Intervention 

The basic design of the LACCDR Project is a pretest–posttest with comparison group design [18]. A list of candidate communities for the project was developed with the Los Angeles County Department of Public Health’s Service Planning Area (SPA) leadership, and other community leaders. Through this process the Steering Committee identified communities that fit the following criteria: (1) Modest-size population, ideally under 50,000 persons; (2) Shared identity as a “community” with at least two of the following: local business community; school/school district; police and fire department services; community clinic/hospital/ health responsible entity; engaged community based organizations; (3) Sufficient community organizational infrastructure to lead local knowledge and capability development and implement LACCDR (*i.e*., via neighborhood planning group); and (4) Diversity in risk exposure and culture/ethnicity. This list was submitted to the Steering Committee who narrowed it down to 16 communities (2 per SPA) that were matched on demographic and hazard risk characteristics (Table 1). Finally, the team’s statistician randomly assigned the two neighborhoods in each of the eight SPAs, for a total of 16 communities, to the intervention or control condition. This entire process lasted from September to December 2012.

**Table 1:** Description of the Sixteen Communities and Coalitions in the Los Angeles County Community Disaster Resilience Project (LACCDR) Project.

Community	Population	Race/Ethnicity	Median Household Income	Percent Renters	Participating Organizations
Acton & Agua Dulce	10,938	His/Lat:18.1% White: 75.9% Afr. Amer: 1.0% Asian: 2.0%	Acton: $87,896; Agua Dulce: $97,000	10.8%	Town Committees, Sheriff’s Department, CERT, U.S. Forest Service
La Cresenta	19,653	His/Lat: 11.4%, White: 57.9%, Afr. Amer: 0.7% Asian: 27.2%	$83,048	35.6%	Fire Safe Committee, Chamber of Commerce, CERT, Fire and Sheriff’s Departments, Assisting Seniors Through Enhanced Resources (ASTER)
Pomona	149,058	His/Lat: 70.5%, White: 12.5%, Afr. Amer: 6.8% Asian: 8.3%	$50,893	44.9%	Emergency Manager, Pomona College, Chamber of Commerce American Red Cross, City Youth and Family services, Police, Tri-city Mental Health
Pico Union	44,664	His/Lat: 66.4%, White: 9.1%, Afr. Amer: 6.2% Asian: 16.5%	$26,424	89.4%	Neighborhood Committee, Police, Fire, County School District, Elementary Schools, Neighborhood Watch, Prevencion y Rescate, Salvation Army, Health Center, Pueblo Nuevo, Kolping House, Latino Community Chamber of Commerce
Culver City	38,883	His/Lat: 23.2%, White: 48.0% Afr. Amer: 9.2% Asian: 14.5%	$75,596	45.7%	Culver City Coalition, Westside Children’s Center, Sony Pictures Entertainment, City School District, Open Paths Counseling Center, Kids are 1st, Medical Center
Watts	51,223	His/Lat: 73.4%, White: 0.6% Afr. Amer: 24.8%, Asian: 0.2%	$25,161	60.0%	Watts Gang Task Force, Kaiser Permanente, City of LA, Concerned Citizens of South Central Los Angeles, Housing Authority, The Center of Grief and Loss
Huntington Park	58,114	His/Lat: 97.1%, White: 1.6%, Afr. Amer: 0.4% Asian: 0.6%	$35,107	73.0%	Community Development Corporation, Fire, Police, American Red Cross, Head Start, Salvation Army, Chamber of Commerce
Wilmington	53,815	His/Lat: 88.8%, White: 4.7% Afr. Amer: 2.7% Asian: 2.0%	$37,277	60.4%	Tzu Chi Clinic, Hubbard Christian Center, Chamber of Commerce, American Red Cross, G.A.P. (Gang Alternative Program), Veterans of Foreign Wars, The Wilmington Teen Center, Philips 66 Refinery, Women of Wilmington, Port Police
Quartz Hill	10,912	His/Lat: 24.6%, White: 62.3%, Afr. Amer: 6.9%, Asian: 2.6%	$56,070	30.4%	Town Committee, Fire, Sheriff’s Department, County School District, Water Board, CERT
San Fernando	23,645	His/Lat: 92.5%, White: 5.3%, Afr. Amer: 0.6%, Asian 0.8%	$52,021	45.5%	City Parks and Recreation, State University Police Services, Valley Care Health System, Mission Community Hospital, Partners in Care Foundation, Police, Public Works, Providence Health Services
San Gabriel	39,718	His/Lat: 25.7%, White: 11.4%, Afr. Amer: 0.8%, Asian: 60.4%	$57,666	50.8%	La Casa de San Gabriel Community Center, Asian Youth Center, Fire, Hope Christian Fellowship, First Presbyterian, St. Anthony’s, Church of our Savior
Hollywood	27,434	His/Lat:: 32.1% White: 48.7% Afr. Amer: 7.6% Asian: 8.1%	$31,415	96.0%	Neighborhood Committees, Hollywood United (H.U.N.K.), United Methodist Church, American Red Cross
Palms	57,964	His/Lat: 29.7%, White: 36.8%, Afr. Amer:10.1%, Asian: 18.9%	$60,728	78.8%	Fire, Police Department, American Red Cross, Community Police Advisory Board (CPAB)
Compton	96,455	His/Lat: 65.0%, White: 0.8%, Afr. Amer:32.1%, Asian: 0.2%	$43,311	44.8%	PACRED churches, Sheriff’s Department, Compton Unified School District, Compton Office of Emergency Management, YWCA.
Hawaiian Gardens	14,254	His/Lat: 77.2%, White: 7.3%, Afr. Amer: 3.4%, Asian: 10.5%	$42,898	55.7%	Emmanuel Church, Celebration Christian Center, Fire Department, City of Hawaiian Gardens, School District, City Committeeman
Gardena	58,829	His/Lat: 37.7%, White: 9.3%, Afr. Amer: 23.9% Asian: 25.8%	$46,961	52.1%	South Bay Coalition for the Homeless, Police Department, CERT, Asian Community Center, Baptist Church

**Figure 1:**
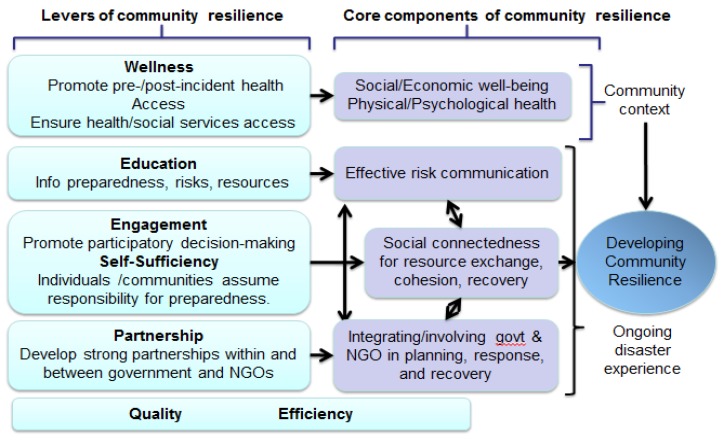
Conceptual Framework for LACCDR (reprinted with permission from Chandra* et al.* 2013) [7].

The conceptual framework for the intervention comes from the work of Anita Chandra and colleagues [7], the Los Angeles County Department of Public Health, and community members. As illustrated in Figure 1, they identified eight essential levers for creating community disaster resilience: wellness, access, education, engagement, self-sufficiency, partnership, quality and efficiency. The LACCDR Project is structured on four of these levers: education, engagement, self-sufficiency, and partnership. Education ensures ongoing information about preparedness, risks and resources before, during, and after a disaster. Engagement involves including community members and promoting participatory decision making in planning, response and recovery activities. Self-sufficiency refers to enabling and supporting individuals and communities to assume responsibility for their preparedness. Organizational partnership involves increasing and enhancing the linkages and collaborations between government and non-governmental organizations (NGOs) and between NGOs in the community. 

**Figure 2:**
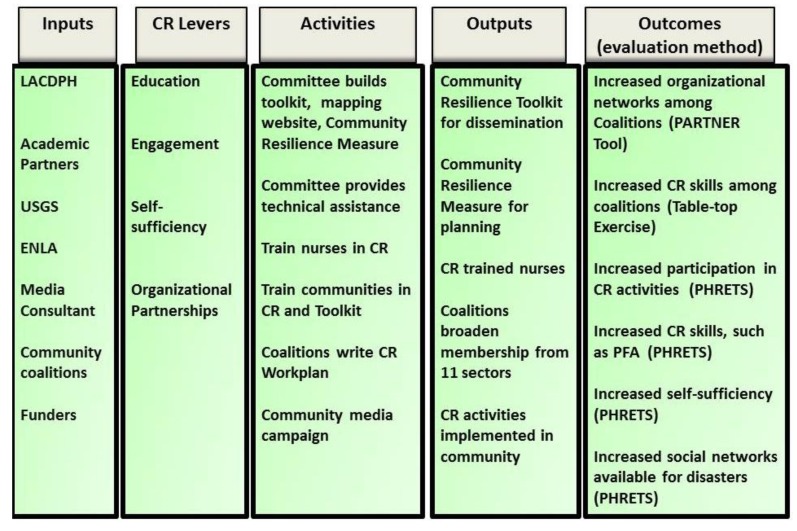
Logic Model for LACCDR.

The Logic Model shown in Figure 2 guides the Project. The outcomes are a result of outputs including nurses trained in CR, a practical CR-building toolkit, and community-based activities led by the coalitions for building their communities’ resilience. The outputs come from operationalizing the levers of resilience in the Chandra model (Engagement, Partnerships, Education, and Self-Sufficiency) through specified activities by the partners and community coalitions using inputs from CDC and funders, the Steering Committee themselves, LACDPH, and the community coalitions. The Outcomes listed in the Logic Model constitute metrics tracked and reported in the evaluation.

Communities randomly assigned to the community resilience group (CR) each have a public health nurse assigned to work with the existing local neighborhood organization to develop a CR coalition dedicated to improving their community’s resilience following community engagement principles. The CR public health nurses are trained in community resilience topics. These topics are assembled into a Toolkit, which is a set of strategies and materials that the community coalitions can use to build resilience in their communities (Table 2). The toolkit developed based on findings from the LACCDR Development Phase includes components deemed a high priority by LA County stakeholders [17]. The modules include sections on how to map the hazards and methods in a neighborhood using web-based tools and other resources; identification of at-risk or vulnerable populations in the community; understanding how to identify and respond to social and psychological trauma; and understanding how to utilize CR trained field workers. The toolkit is designed to be interactive with questions and activities to generate community-specific discussions. For instance, in one module each coalition conducts a community hazard assessment exercise with the goal of prioritizing the hazards on which to focus. 

A novel resource developed specifically for the LACCDR Project is mapping software called Sahana (Sahana Software Foundation) that allows communities to map their sources of risk and resilience [19]. Communities in the resilience arm have access to training in and use of mapping and charting software that allows them to visualize the relationships between local hazards, socio-demographic subsets of their community (with a focus on vulnerable populations), and assets and resources available to the community (community organizations, CERT trained individuals, evacuation routes). Through mapping, CR communities can map which hazards may affect their community to support planning and prioritization, assess the relationship between hazards and vulnerable populations, and incorporate the roles of community-based organizations, faith-based organizations, government agencies and other sectors that are local resources to be engaged in their resilience planning. Sahana is an open source disaster management software that features the capability to display and overlay maps, manage resources, register organizations, and conduct surveys. Hazard maps, census data, and resource datasets were developed and loaded into Sahana for each of the eight CR communities. Hazard maps were provided by the United States Geological Survey Science Application for Risk Reduction program. Resource data came from the Los Angeles County Resource Listing available on the Los Angeles County GIS Data Portal [20]. Sociodemographic data were downloaded from the American Community Survey 2010. Specific functionalities were built to allow community members to collect and input further data on hazards, organizations, and community events. The website was built with membership from the coalitions advising through a user’s group. All nurse facilitators in the CR arm and members of the eight coalitions received training in using Sahana. Ongoing technical assistance is available from Sahana to the coalitions.

**Table 2:** Description of Community Resilience Toolkit in LACCDR Project.

Toolkit Section	Description (and Levers Addressed in Section)
Psychological First Aid	Psychological First Aid is designed to reduce disaster-induced stress by prompt provision of social support, linkage to resources, and promotion of effective coping strategies and coping self-efficacy. (Levers: Education, Self-Sufficiency)
Community Mapping	Community mapping is a process to identify resources and develop connections among people and their local organizations. There are several options for engaging in this process that vary in their use of technology, scope, and scalability. An aspect of the community mapping activity is helping communities consider access and functional needs populations. (Levers: Education, Self-Sufficiency, Engagement)
Community Engagement Principles for CR	A set of community engagement principles are applied to CR building initiatives and are applicable to responder agencies, community and faith-based organizations, community leaders and stakeholders, and community members. (Levers: Education, Self-Sufficiency, Engagement, Organizational Partnerships)
How to Identify and Develop Community Leaders	This section provides supports for communities to have effective leadership for CR. (Levers: Engagement, Organizational Partnerships)
Training Community Field Workers	Guidelines and resources for CR field workers, including nurses, school staff, and lay community health workers to support CR in communities. Includes a curricula on disaster preparedness. (Levers: Education, Self-Sufficiency, Engagement)

The coalitions meet monthly in their communities and the CR public health nurses lead their coalitions through training in the Toolkit, with technical assistance from Steering Committee members as requested. Based on this process, each CR coalition develops a written CR Workplan for improving community resilience in their neighborhood. The coalitions choose a yearlong scope of work in the CR Workplan. The CR Workplan is informed by their trainings in the Toolkit sections and knowledge of their local priorities and is intended to build on the assets and partnerships existing in the coalition and in the community. Using the CR model and toolkit sections is not required but is encouraged with direction and support from the public health nurses and Steering Committee members. The “Community Resilience Measure” created specifically for this project is a tool meant to facilitate the translation of the CR model into their CR Workplans. The Community Resilience Measure guides the coalitions in creating a plan that operationalizes CR principles from the Chandra model (see Table 3). Each coalition’s CR Workplan is shared with the Steering Committee who provides a review based on the Community Resilience Measure and recommendations for enhancing the CR Workplan so that it addresses individual items in the Community Resilience Measure. Once the CR Workplan is reviewed and approved by LACDPH in conjunction with projected expenditures, the coalition receives $15,000 in funding to use in implementing their plan.

The comparison group has a public health nurse or health educator assigned to develop a Preparedness coalition. In the comparison arm, the public health nurse or health educator takes a traditional educational approach by training the coalitions using a standardized, organized manual of public health practice for improving disaster preparedness [21]. (Disaster preparedness is conceptualized as focusing mainly on personal or household self-sufficiency through accumulation of supplies and emergency communication plans). Training topics include individual and family preparedness; considerations for special populations such as kids, animals, special needs, and seniors; communication tools; and linking with local nonprofits, faith-based organizations, and small businesses. The public health nurses or health educators who serve this function are not trained in community resilience and neither the public health workers nor the communities participating in the comparison arm have access to the CR toolkit or subject matter experts for technical assistance. Preparedness coalitions also create a written workplan and once approved, receive $15,000 to implement their activities.

## 3. Evaluation

Evaluation of outcomes of the LACCDR Project aims to identify improvements in indicators of community resilience at multiple levels. The project uses a mixed methods evaluation strategy designed to focus on changes in community organization relationships, population practices and awareness, and evidence of change in the coalitions’ skills and understanding of CR. These are being measured by an organizational network survey, a population-based survey, and table-top exercises with the coalitions, respectively. Specific descriptions follow below.

**Table 3:** Community Resilience Measure.

Thinking about Your Community’s Plan Overall, Please Answer the Following Questions:
**Priority Vulnerable Community Members** (Levers addressed: Engagement, Organizational Partnerships) Who are your most vulnerable community members? What mapping tool or other processes are you using to identify those vulnerable community members and where they are concentrated? What are the limitations these vulnerable community members have in either mobility, communications or resources that make them particularly vulnerable in a disaster? How are you including those vulnerable members in your planning process? (Planning “with” not “for” them.) What are the assets, resources, and networks that vulnerable community members already have and how are you using them in your resilience plan?
**Understanding Your Community ** (Levers addressed: Engagement, Organizational Partnerships, Self-Sufficiency) What mapping tool or other process are you using to identify the hazards in your community? What mapping tool or other process are you using to identify your community’s resources? How are you using the information you collected to get your neighbors and your community prepared, ready to respond, and able to recover from a disaster or emergency? How are you encouraging neighbor to neighbor discussion or planning to support one another in a disaster or emergency?
**Important Sectors in your Community ** (Levers addressed: Engagement, Organizational Partnerships, Self-Sufficiency) How are you getting organizations and agencies from the CDC 11 sectors involved in the coalition? List the types of organizations you’ve identified (those you already have, those you wish to still bring onboard)? What roles do they play in the coalition (*i.e*., leading or supporting activities)? How are you using the services and resources that these organizations and agencies bring in your community? How are you coordinating the work of first responders and community members to avoid overlap and keep information flowing and lines of communication open?
**Recovery ** (Levers addressed: Organizational Partnerships, Self-Sufficiency) How are you planning to help families, neighborhoods, and the community as a whole recover? How will organizations and agencies in your community continue to help their current clients as well as the wider community, too? How are organizations and agencies in your community involved in planning for the recovery process?

### 3.1. Organizational Network Analysis

As discussed above, one of the theoretical levers for changing CR is increasing organizational partnerships by increasing and deepening the linkages among community NGOs. To assess improvements in this domain, the Project is measuring longitudinal changes in inter-organizational linkages among NGOs in the 16 communities. This evaluation seeks to determine if the coalitions in the community resilience arm improve their partnerships with community organizations, as measured by increases in their organizational linkages, compared to coalitions in the comparison group. The Project uses a social network analysis tool called PARTNER (Program to Analyze, Record, and Track Networks to Enhance Relationships) at the start of study and at least once more during the study period [22]. Data collected by the project will determine the quality of relationships among partners, how they change over time, and examine how they are leveraged to achieve resilience outcomes in eight communities in LA County. PARTNER is a software program that consists of a brief survey linked to an analysis tool that visually maps the collaborative network and analyzes the number, strength, and quality of connections among partners. This tool provides a means for measuring the process of exchange and interaction among participating organizations in a coalition over time and the activities in which each coalition is engaged.

Partnerships are measured primarily by connectivity. Connectivity is defined as the measured interactions between partner organizations in the coalition, such as the amount and quality of interactions, and ways in which these relationships change over time [22]. The following measures are used to assess connectivity: Types of relationship (e.g., is there information sharing, joint program development, or resource exchange?); trust among partners (measured as an index of reliability, mission agreement, and ability to have open discussion around issues); and value of the partner organizations to the coalition’s mission (measured as power/influence, commitment, and resources provided). 

Two levels of community resilience outcomes will also be measured, intermediate capacities of the coalitions and their final outcome capabilities. Intermediate capacities are necessary for the coalition to work together to achieve its goals of improving community resilience. Coalition capacities include having completed an assessment of vulnerabilities and assets, having a plan for delivering psychological first aid in an emergency, and recruiting volunteers for coalition activities. In addition, coalitions should impact community resilience capability outcomes. These outcomes describe the capacity of the system to provide both routine and emergency services. Capabilities include coalition members participating in exercises and drills, the community exercising its plan in an actual emergency, and the community has closer ties to the Los Angeles County Department of Public Health.

### 3.2. PHRETS Household Survey

An outcome indicator of the Education, Self-Sufficiency and Engagement levers is longitudinal changes in neighborhood residents’ community resilience activities and attitudes. A survey measures the resilience-related awareness, attitudes and practices of residents in the sixteen Los Angeles County communities. The survey is a repeated cross-sectional design conducted in English, Spanish and Korean languages. 

Because the research design involves distinct treatment and control communities it is essential to select a sample of each of the sixteen communities, and for each sample element to be geographically linked to one and only one of those sixteen communities. For that reason, address-based sampling is being used. A sample size of N = 4400 participants at each time point (n = 2200 per arm) provides enough power to detect 4.22% change in two proportions without adjusting for clustering and 8.74% change in two proportions adjusting for clustering (ICC = 0.01). Survey domains include: household preparedness for disaster; participation in community resilience building activities; self-efficacy for helping in a disaster; perceived collective efficacy of the community in a disaster; perceived benefits of individual preparedness and perceived benefits of disaster planning with neighbors; locus of responsibility; trust in public health in a disaster; social networks available in a disaster; civic engagement; social cohesion; self-reliance in a disaster; perceived health and activity limitations; and demographics. The survey domains were selected as outcome indicators for the theoretical levers. For example, outcomes in the self-sufficiency domain can be measured by changes in household preparedness, self-efficacy for helping in a disaster, and perceived collective efficacy of the community in a disaster. Outcomes in the engagement domain can be measured by changes in participation in community resilience building activities. Outcomes in the education domain can be measured by changes in perceived benefits of individual preparedness and perceived benefits of disaster planning with neighbors.

### 3.3. Table-Top Exercise

While the organizational network and community resident surveys are key to understanding practice, attitudes, and potential changes in resilience approaches, it is difficult to assess how a community or coalition may act in an actual event. In order to simulate an event condition and assess the extent to which coalitions were strengthening the four CR levers (engagement, self-sufficiency, partnership, and education), the Project developed and will conduct a series of CR tabletop exercises. The CR tabletop is built on a traditional tabletop design, used for events such as pandemic influenza [23], but is unique in its testing of how coalitions will work together to leverage community assets, address the needs of vulnerable populations, integrate government and NGO roles and plans, and ensure community ability to recover over the long-term. In order to test resilience principles, the table-top employs a scenario that is seemingly modest at start (a heat wave) but then escalates over time with other changes in community conditions (crime increases, drought worsens, brown-outs occur, and community members die). As designed, this allows the coalition to consider the extent and quality of their partnerships and assets for an expansive and lengthy event rather than what is traditionally tested in tabletops, that is, a massive, acute scenario. 

The tabletop is designed to be relatively brief at two hours. The presentation of the scenario with prompts and two unfolding situations lasts 1.5 h. The debriefing and discussion that follow take 30 min. Participants are asked to rate how they responded during the scenario along the four levers. For example, participants are asked to rate their response on a scale of 1–5 for partnership, with 1 indicating that they have very little awareness of the sectors to bring into planning and response, and 5 noting all sectors are engaged and fully integrated into the response and recovery plan and that government and NGO is working collaboratively. A similar scale is used for the other three levers. In addition to coalition member ratings, the research team provides their own independent ratings based on observations. After the coalition concludes the tabletop, the study team provides a brief summary of their discussion. For the purpose of evaluation, the same tabletop exercise is conducted across all sixteen coalitions. However, the CR coalitions receive a more expansive summary with recommendations for action steps to improve their resilience responses (e.g., ideas and strategies for improving partnerships, ideas for considering the assets they need for recovery, considerations for the psychological and social impacts of long-term recovery), while the comparison coalitions only receive a brief summary of their discussion with no recommendations or insights from the study team. The study team then works with each CR coalition to consider the gaps or areas for improvement identified in the tabletop, with the goal of addressing those gaps over the next study year. The tabletop will be administered at two time point to assess change over time. 

### 3.4. Process Evaluation

The project is also conducting a process evaluation using a mix of qualitative and quantitative methods (ethnography, participant reflections, document review) and measures of reach into the community. The process evaluation is examining the factors that promote development and impact of community coalitions as agents of change towards resilience. The process evaluation aims to describe how coalitions adopt resilience based principles and develop and implement community disaster resilience plans, including facilitators and barriers to these processes. During the coalition meetings, the evaluation team collects data that documents what the coalitions are doing and to what extent they are moving forward with respect to the four levers of resilience in the Chandra model. Observers attend meetings and take notes to capture how the 16 coalitions have: interpreted, adopted and adapted the information they have received; addressed the need for engagement of organizations and vulnerable populations from outside of their coalitions; discussed how to establish robust partnerships with other organizations/sectors within their communities; contemplated how to leverage their existing resources in efficient ways; and, considered how to plan at the community level rather than at the individual level. In addition, participants are asked to provide feedback through “Reflection Sheets” on successful and challenging elements of the meeting and toolkit training, raise any questions that they have about the Project, and suggest additional issues that they want addressed in the future. 

## 4. Discussion and Conclusions

The LACCDR Project will shed light on several issues in the effort to build community resilience. We will have gained experience using community engagement and encouraging governmental and non-governmental partnerships as an approach to increasing resilience. We will learn more about training public health nurses in community resilience approaches, explaining resilience to community members, and what technical assistance is needed to be successful. We may learn how existing expertise and resources in our department of public health, collaborating organizations, and community coalitions can be leveraged to develop effective strategies that build communities that are more resilient to disasters. Finally, we will have data on the outcomes of a resilience building initiative and how it differs from the outcomes of a traditional method that focuses solely on household preparedness and does not actively promote community engagement and broad partnerships. The tools and measures we developed will be useful to similar efforts across the nation even as every community differs in leadership structure, assets, and other situational variations that require a different application of the tools we have developed and tested.

The LACCDR Project has limitations and challenges. A major limitation is constraints on measuring community resilience, a problem well known to practitioners in the field [24]. Project findings must be treated with caution as a result. A major challenge is that public health lacks tested tools for building community resilience. The process evaluation will provide important information for improving the toolkit so we expect it to be revised along the way. There will be variability among the communities in how they respond to the program because of differences in assets, resources, partnerships and leadership. Since much of community resilience is reflected in these differences, some degree of improved community resilience outcomes should be expected in both arms of the study.

The LACCDR Project is unusual in the United States because it implements and evaluates a public health led program for increasing community disaster resilience. Community resilience building activities can improve overall social cohesion and important aspects of community well-being so the implications of this study extend beyond the disaster preparedness area. It is therefore useful as a model for operationalizing policy directives for improving community disaster resilience and improving general community well-being. The study suggests specific programmatic activities and partnership approaches that can be implemented and evaluated in communities across the country. 

The LACCDR Project is one of several such efforts to improve community resilience in the United States, most of which are led by first responder agencies such as the Federal Emergency Management Agency (FEMA) and the American Red Cross. America’s PrepareAthon, led by FEMA, is a community-based program that aims to increase community engagement in community resilience planning and improve participants’ knowledge of hazards and how to stay safe and mitigate damage [25]. American Red Cross is working on community resilience in many of its programs, some of which include community engagement as a component) [26]. 

However, neither of these efforts employ a systematic assessment of both individual and organizational change. Using findings from the LACCDR Project coupled with these other ongoing activities, state and local public health officers will soon have a more complete roadmap of how to translate high-level policy directives for community disaster resilience building into the implementation and evaluation of resilience building activities in communities.
